# Nitrogen-Fixing Bacteria in *Eucalyptus globulus* Plantations

**DOI:** 10.1371/journal.pone.0111313

**Published:** 2014-10-23

**Authors:** Marliane de Cássia Soares da Silva, Thiago de Almeida Paula, Bruno Coutinho Moreira, Manuela Carolino, Cristina Cruz, Denise Mara Soares Bazzolli, Cynthia Canedo Silva, Maria Catarina Megumi Kasuya

**Affiliations:** 1 Departamento de Microbiologia, Universidade Federal de Viçosa, Viçosa, Minas Gerais, Brazil; 2 Faculdade de Ciências da Universidade de Lisboa, Centro de Biologia Ambiental, Lisboa, Campo Grande, Portugal; Agroecological Institute, China

## Abstract

Eucalypt cultivation is an important economic activity worldwide. In Portugal, *Eucalyptus globulus* plantations account for one-third of the total forested area. The nutritional requirements of this crop have been well studied, and nitrogen (N) is one of the most important elements required for vegetal growth. N dynamics in soils are influenced by microorganisms, such as diazotrophic bacteria (DB) that are responsible for biological nitrogen fixation (BNF), so the aim of this study was to evaluate and identity the main groups of DB in *E. globulus* plantations. Samples of soil and root systems were collected in winter and summer from three different Portuguese regions (Penafiel, Gavião and Odemira). We observed that DB communities were affected by season, N fertilization and moisture. Furthermore *Bradyrhizobium* and *Burkholderia* were the most prevalent genera in these three regions. This is the first study describing the dynamic of these bacteria in *E. globulus* plantations, and these data will likely contribute to a better understanding of the nutritional requirements of eucalypt cultivation and associated organic matter turnover.

## Introduction

*Eucalyptus globulus* Labill. occurs naturally in Tasmania and southwest Australia and was introduced into Portugal in the 1950s. The genus *Eucalyptus* belongs to the family Myrtaceae and is comprised of a variety of species distributed throughout the world and grown under a wide variety of environmental conditions [1]. Eucalypts are cultivated primarily for the production of cellulose, paper, boards, plywood sheets, charcoal and pharmaceuticals [1]. It is estimated that in 2030, due to strong demand, annual lumber consumption by industries will reach 2.44 billion m^3^, which represents a 45% increase over consumption in 2005 [2]. The rapid growth of eucalypts demands high availability of soil nutrients, especially nitrogen (N), which is absorbed and accumulated in large quantities [3], [4]. The productivity of eucalypt forests is directly related to the nutrient balance of the soil.

Nitrogen in the atmosphere (N_2_) is the primary N reservoir and can be made available through the process of biological nitrogen fixation (BNF). Due to their high energy costs, BNF processes are restricted to microorganisms that have the nitrogenase enzyme complex [5]. These N-fixing microorganisms can be free-living, associative or mutualistic [6], [7].

The association of diazotrophic bacteria (DB) with different plant species has been detected using various microbiological and molecular biological techniques [8]–[10]. Studies of these bacteria represent new ways of understanding the dynamics of the microbial communities involved in N cycling, especially with the use of genes that encode enzymes involved in BNF. The *nifH* gene, which encodes nitrogenase reductase, is an example of these genes [8], [11], [12].

The nested polymerase chain reaction with denaturing gradient gel electrophoresis (Nested-PCR-DGGE) techniques using the *nifH* gene is an efficient tool for detecting microorganisms that are directly related to N cycling. DGGE can monitor changes in the community structure in response to changes in environmental parameters [10], [13]–[16].

Despite the commercial and economic importance of eucalypts, there are very few studies of the microbial communities in their ecosystems, and little is known of how soil microorganisms interact with and benefit eucalypt N nutrition. Furthermore, the adaptation of eucalypts to different environments could be related to their interactions with soil microorganisms, including DB, which have been found to be associated with eucalypt trees in the equatorial climate of Colombia [17]. Thus, our hypothesis is that there are endophytic or free-living DB associated to *E. globulus* in the studied regions, and that these can contribute to eucalypt N nutrition. The aims of this study were to evaluate the DB community's dynamics, identify the main groups of DB in *E. globulus* plantations, and the effects of season and N fertilization on these microorganisms.

## Materials and Methods

### Letter of authorization

In 2010, a cooperation protocol was established between the Institute of Forest and Paper Research (RAIZ Institute) of the Portucel Soporcel Group and FCUL (Science Faculty of the University of Lisbon) in the context of the latter's research project “The importance of cooperation in microbial genomics nitrogen use efficiency – The case of Eucalyptus” coordinated by Dr. Cristina Cruz. In general, the RAIZ pledged to cede some areas to allow field studies necessary for the development of the study, and in return, information of interest related to this project would be provided to RAIZ for its internal research “Dynamic N in forest soils”. The field work was led by the researcher and doctoral student Marliane Cassia Soares da Silva, FCUL, with the support of RAIZ's Lands and Forest Nutrition team and field technicians. Three study sites were selected and sampled over two seasons (winter and summer). Samples from the soil were taken according to the methodology of FCUL. RAIZ supplied information on existing methods to be used in the study, especially with regard to forestry and historical specificities of selected treatments within the trials. Authority responsible: Sergio Fabres (sergio.fabres@portucelsoporcel.com) and Daniela Ferreira: (daniela.ferreira@portucelsoporcel.com).

### Study site

This study was carried out in *E. globulus* plantations owned by the Portucel-Soporcel Group, in three regions of Portugal: Penafiel in the north, Gavião in the central and Odemira in the south region. To realisation, this study an accord of cooperation was established between the Portucel-Soporcel Group and Faculty of Science, University of Lisbon. These plantations were about 8 years old. The three regions were selected due to their distinct soil physicochemical properties, rainfall and climate (Table 1). In January (winter) and June 2011 (summer), samples of soil and root systems were collected from two plots within each plantation, which both had received the initial N fertilization (3.3 g N per plant) upon being planted, and only one plot from each plantation had then received N maintenance fertilization (60 kg N ha^−1^y^−1^) in the period 2004–2007. So, six plots were sampled.

**Table 1:** Soil characteristics, rainfall and climate for the *Eucalyptus globulus* in the Penafiel, Gavião and Odemira regions.

Region	Textural Class		FAO Classification (1998)		Lithology	Stony (%)	Average Rainfall annual (mm)	Winter	Summer	Average Temperature annual (°C)	
Penafiel	Sandy loam		Regosols epileptic		Sediments	10%	1500	Fresh	Moderate	12.8	
Gavião	Sandy loam		Regosols epileptic skeletic		Shale and Grauvaques	5%	850	Moderate	Hot	9.7	
Odemira	Sandy loam		Umbrisol endoleptic arenic		Granite	50%	750	Moderate	Hot	14.6	
	**N**	**P**	**K**	**Ca**	**Mg**	**Na**	**B**	**Cu**	**Zn**	**Fe**	**Mn**
	**g Kg^−1^**	**mg Kg^−1^**	**cmol Kg^−1^**				**mg Kg^−1^**				
Penafiel	1.3	4.8	0.09	0.08	0.05	0.07	1.0	0.3	0.7	168	4.8
Gavião	1.12	0.4	0.6	0.77	0.4	0.03	0.5	1.8	1.3	52	0.6
Odemira	2.25	7.0	0.14	0.58	0.48	0.22	1.0	0.3	0.7	41	61.6

### Sampling

Three composite samples were collected from each plot. Each composite samples consisting of soil or roots collected from three neighbouring trees. In each tree, ten random points were sampled in a radius of 40 cm around the trunk at a depth of 0–10 cm. The fragments of roots were composed primarily of fine roots (0–1 mm in diameter). The samples were placed in plastic bags and stored on ice, transported to the laboratory, and then stored at −20°C for up to two months before analysis.

### Soil characterization

Soil samples were classified according to the FAO/UNESCO (Table 1). These samples were weighed and placed in an oven at 60°C for 48 h to determine moisture content.

Organic matter content was determined using the loss-on-ignition method. One gram of soil was weighed in a porcelain crucible and placed in a muffle furnace (Nabertherm 30–3000°C, model L3, Germany) at 500°C for 12 h [18].

Soil extracts were prepared in potassium chlorate (2 mol L^−1^) using 2 g of soil for pH determination in distilled water made up to a final volume of 20 mL. A second soil extract was prepared by placing 2 g of soil from each sample in distilled water made up to a final volume of 20 mL, then incubating the mixture under constant agitation for 1 h. A 10 mL of this extract was then centrifuged and used to determine the ammonia concentration by the colorimetric method [19]. Absorbance was measured on a microplate reader (Spectra Rainbow – TECAN) at 665 nm.

### Urease activity

Urease activity in the soil was determined using the colorimetric method according to Kandeler and Gerber [20]. Assays were performed in triplicate with one gram of soil for each composite samples and absorbance were determined on a microplate reader (Spectra Rainbow – TECAN) at 665 nm.

### Determination of nitrification potential

The nitrification potential of ammonia-oxidizing bacteria was determined using 15 g of fresh soil exposed to an ammonium sulfate solution. The accumulation of nitrite over a 6 h incubation period was then measured. Three replicates were performed for each composite samples. The soil was incubated for 6 h at 27°C at 760 rpm constant agitation in a solution containing ammonium sulfate, dibasic potassium phosphate, monobasic potassium phosphate and sodium chlorate to inhibit nitrite oxidation (according to the ISO 15685 standard). The reaction was stopped with potassium chlorate and the samples were centrifuged; the nitrite concentrations were then determined using solutions of sulfanilamide and N-(1-Naphthyl) ethylenediamine. The absorbance was determined on a microplate reader (Spectra Rainbow – TECAN) at 545 nm.

### Isolation and quantification of diazotrophic bacteria

The number of DB was quantified by plate counts on LGI culture medium [21]. Ten grams of soil from composite samples were placed in an Erlenmeyer flask containing 90 mL of saline solution (0.9% w/v of sodium chlorate) and kept under constant agitation at 760 rpm for 1 h. Serial dilutions were then performed in saline solutions up to 10^−6^, and 0.1 mL of each dilution was plated on LGI medium. The analyses were conducted in triplicate.

### Nested-PCR of *nifH* genes

Total DNA was extracted from 250 mg of the root system and soil samples using an *UltraClean PowerSoil* kit (Mobio Laboratories, *Solana Beach, CA*, USA) according to the manufacturer's instructions.

The total DNA was used as template in a PCR reaction to amplify the *nifH* gene, which is involved in the N fixation process. The primers 19F and 407R used to amplify *nifH*, producing a 390 bp fragment, as described by Ueda et al. [22]. This step was followed by nested-PCR using the oligonucleotides 19F-C (with addition of the GC-clamp) and the primer 278R [24], which yields a 260-bp fragment. The PCR mixture consisted of 20 ng of total DNA, 0.2 µM of each oligonucleotide, 200 µM dNTP, 2 mM MgCl_2_, 0.5 mg mL^−1^ bovine serum albumin (BSA) and 1.25 units of GO Taq DNA polymerase (Promega, Madison, USA) in a total reaction volume of 50 µL. PCR reaction cycling conditions were those described by Direito and Teixeira [23].

### DGGE

The DNA fragments obtained using the nested-PCR technique on the soil and fine root samples were analysed by DGGE (DCode System, Bio-Rad Inc., California). DNA extracted from pure cultures of DB of the genera *Pseudomonas fluorescens*, *Bradyrhizobium* sp., *Bradyrhizobium elkanii*, *Bradyrhizobium japonicum*, *Rhizobium tropici*, *Burkholderia brasiliensis*, *Burkholderia sabiae*, *Stenotrophomonas maltrophila*, and *Acinetobacter calcoaceticus* were used as templates in nested-PCR, and the amplicons obtained were used as external markers. A 20 µL sample of the nested-PCR products ranging from 150 to 200 ng of DNA was loaded onto an 8% (w/v) polyacrylamide gel in 1 X Tris-acetate-EDTA (TAE) buffer. The gel was prepared with a denaturing gradient ranging from 50% to 65% (where 100% denaturation indicates a concentration of 7 mol L^−1^ urea and 40% formamide). The gel was subjected to vertical electrophoresis at 60 V for 12 h at 60°C, then stained for 40 min with *Sybr Gold* (1x) (Molecular Probes, Leiden, The Netherlands); the gel was then photographed under UV light on a Molecular Imaging System (Loccus Biotecnologic L-Pix Chemi). The bands of interest were excised, eluted, used as templates in a new PCR reaction using the same primers but without addition of the GC-clamp [24], and then sequenced by Macrogen, Inc. (Korea). All sequences obtained in the present study were edited using Sequencher software (Version 4.1, Gene Codes Corp., Ann Arbor, USA). The results were analysed by comparing the obtained sequences with those deposited in the GenBank database using the BLASTx search tool (NCBI).

### Statistical analyses

The analyses of soil characterization were made in triplicate. The data were subjected to variance analysis (ANOVA), and mean values were compared using Tukey's test (p<0.05) by Saeg software (version 9.1, Universidade Federal de Viçosa).

The DGGE profiles, aligned based on the external markers, were analysed and compared using BioNumerics software (Version 5.1, Applied Maths NV). The bands were identified and the areas corresponding to each band were quantified relative to total sample area, allowing for normalization and subsequent comparisons between samples. The correlations between the environmental data and the occurrence of DGGE bands and their intensities were determined using Principal Component Analysis (PCA) with Canoco software (version 4.5, Biometris, Wageningen, Netherlands). Distinct bands observed in the DGGE gels were considered to be different species, and their relative intensities were considered to be the frequency at which these species occur. The ammonia, urease, organic matter, moisture and pH results were considered as environmental variables.

## Results

### Soil characterization

Soils were acidic and moisture varied greatly with season and region, reaching very low values in summer, specially in Gavião. Ammonia was present in very small amounts both in winter and summer, even in the areas which received N fertilizer (Table 2). At all regions, the nitrification potential was very low and the urease activity was influenced by the season analysed.

**Table 2:** Physicochemical characteristics of the soils of the studied *Eucalyptus globulus* plantations in the Penafiel, Gavião and Odemira regions.

Region	Treatment	Organic matter (%)		pH		Moisture (%)		Urease activity (µg N-NH_4_ g^−1^ dry soil h^−1^)		Ammonia (µg N-NH_4_ g^−1^ dry soil)	
		winter	summer	winter	summer	winter	summer	winter	summer	winter	summer
Penafiel	WNF	7.57±0.98	9.02±1.56	3.76±0.08	3.85±0.08	16.53±1.95	9.36±0.99	9.48±2.62	19.32±18.19	4.96±8.12	0.00±0.00
	NF	9.42±0.87	9.46±0.76	3.79±0.07	4.04±0.38	14.68±3.91	8.34±2.32	10.49±6.63	8.12±14.06	0.00±0.00	1.09±1.89
Gavião	WNF	5.24±1.38	4.82±0.07	4.17±0.07	4.08±0.10	11.64±2.20	5.75±2.04	1.73±1.81	13.92±6.00	2.25±3.89	2.67±4.62
	NF	7.66±3.12	6.05±0.06	3.75±0.06	3.79±0.17	10.50±1.17	4.49±0.85	0.00±0.00	5.85±8.33	0.00±0.00	0.00±0.00
Odemira	WNF	12.74±0.40	17.50±3.98	3.43±0.17	4.74±1.32	18.22±1.08	15.20±0.75	14.92±7.55	26.06±8.01	3.80±6.58	6.10±6.72
	NF	12.85±0.95	14.09±1.90	3.20±0.06	3.52±0.03	21.04±1.61	15.66±2.73	8.76±2.04	29.29±18.38	0.00±0.00	0.00±0.00

WNF- Without nitrogen maintenance fertilization; NF – with nitrogen maintenance fertilization.

### Quantification of culturable diazotrophic bacteria (DB)

Culturable diazotrophic bacteria were found in all studied areas. However, no influence of region, season or N fertilization on the number of culturable DB was observed (CFU; p>0.05) (Table 3).

**Table 3:** Influence of nitrogen maintenance fertilization and season on Colony Forming Unit (CFU) counts of diazotrophic bacteria in soil samples from *Eucalyptus globulus* plantations in the Penafiel, Odemira and Gavião regions.

Region	Treatment	Log (CFU) g^−1^ dry soil	
		Winter	Summer
Penafiel	WNF	5.26±0.35	5.61±0.14
	NF	5.67±0.09	5.49±0.28
Gavião	WNF	5.57±0.16	5.46±0.40
	NF	5.61±0.15	5.26±0.31
Odemira	WNF	6.03±0.13	6.33±0.19
	NF	5.84±0.10	5.96±0.41

WNF – Without nitrogen maintenance fertilization; NF – with nitrogen maintenance fertilization.

### Analysis of Diazotrophic bacteria communities

**Figure 1:**
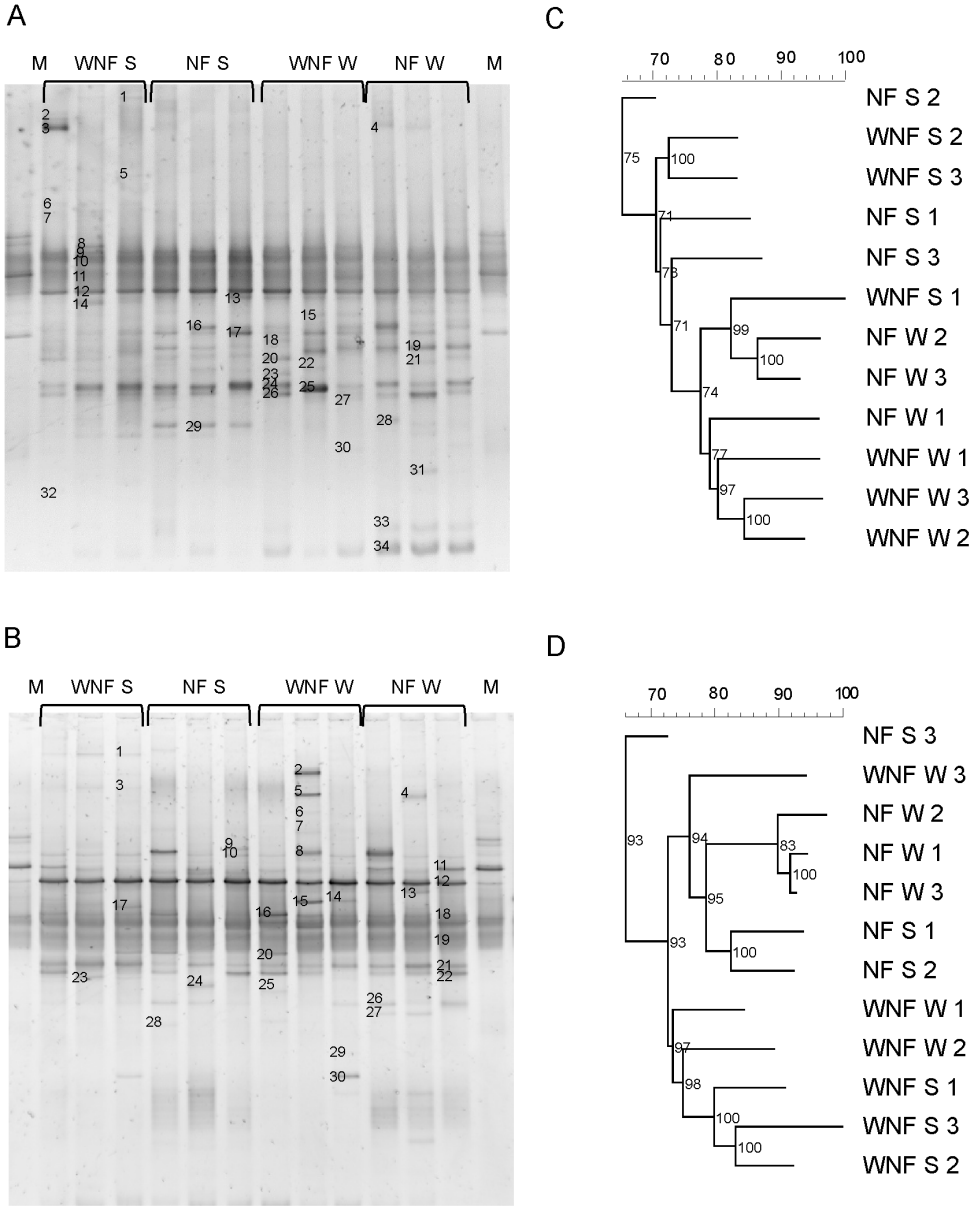
Analysis of the neighbour-joining cluster obtained from the DGGE banding patterns of the microbial communities in soils of *E. globulus* plantations in the Penafiel region of northern Portugal. **A**) DGGE band pattern of soil samples using the *nifH* gene. **B**) DGGE band pattern of root system samples using the *nifH* gene. **C**) Dendrogram of soil samples. **D**) Dendrogram of root system samples. NF: with nitrogen maintenance fertilization; WNF: without nitrogen maintenance fertilization; W: winter; S: summer; M: Marker. Scale  =  similarity (%).

**Figure 2:**
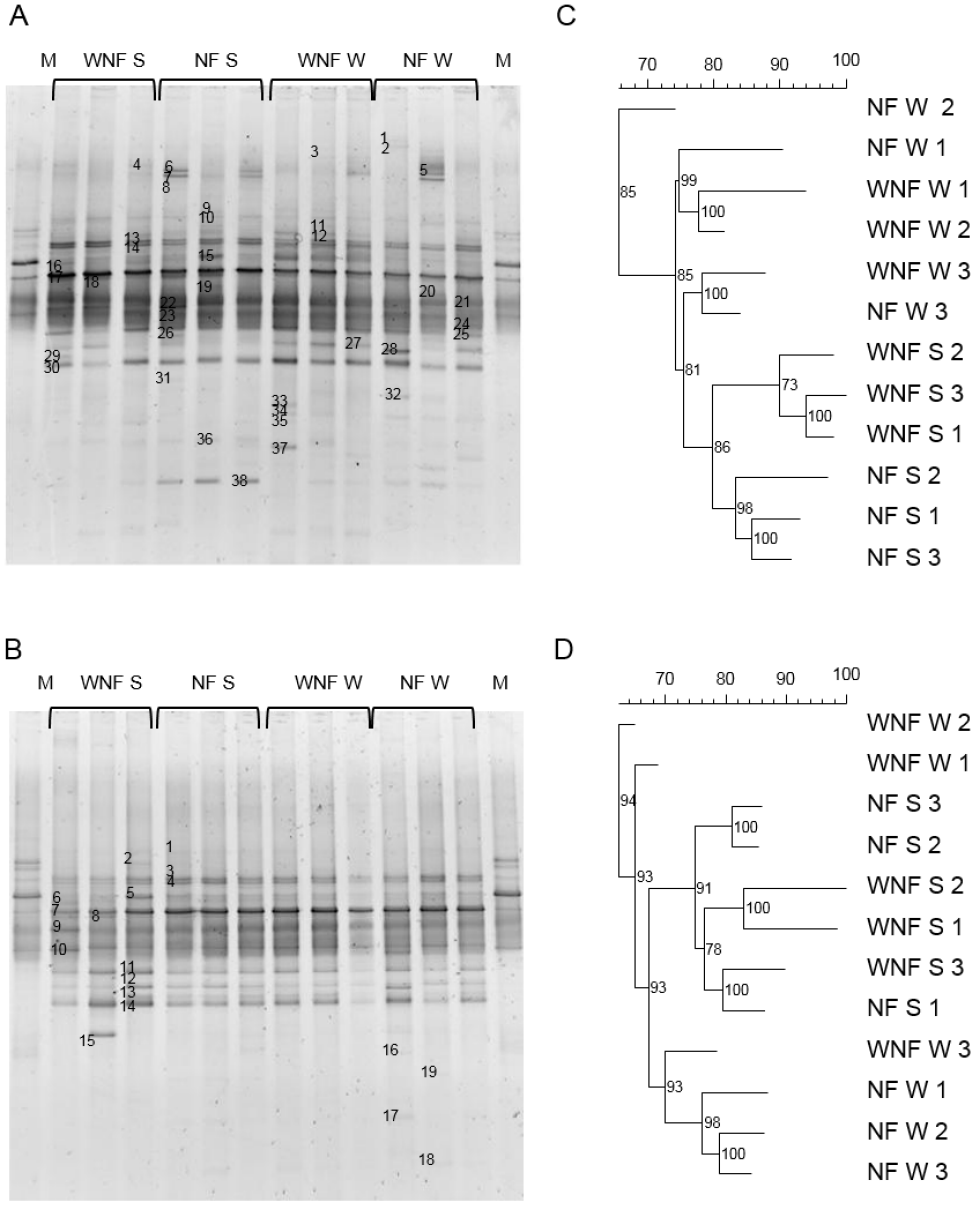
Analysis of the neighbour-joining cluster obtained from the DGGE banding patterns of the microbial communities in soils of *E. globulus* plantations in the Gavião region of central Portugal. **A**) DGGE band pattern of soil samples using the *nifH* gene. **B**) DGGE band pattern of the root system samples using the *nifH* gene. **C**) Dendrogram of soil samples. **D**) Dendrogram of root system samples. NF: with nitrogen maintenance fertilization; WNF: without nitrogen maintenance fertilization; W: winter; S: summer; M: Marker. Scale  =  similarity (%).

**Figure 3:**
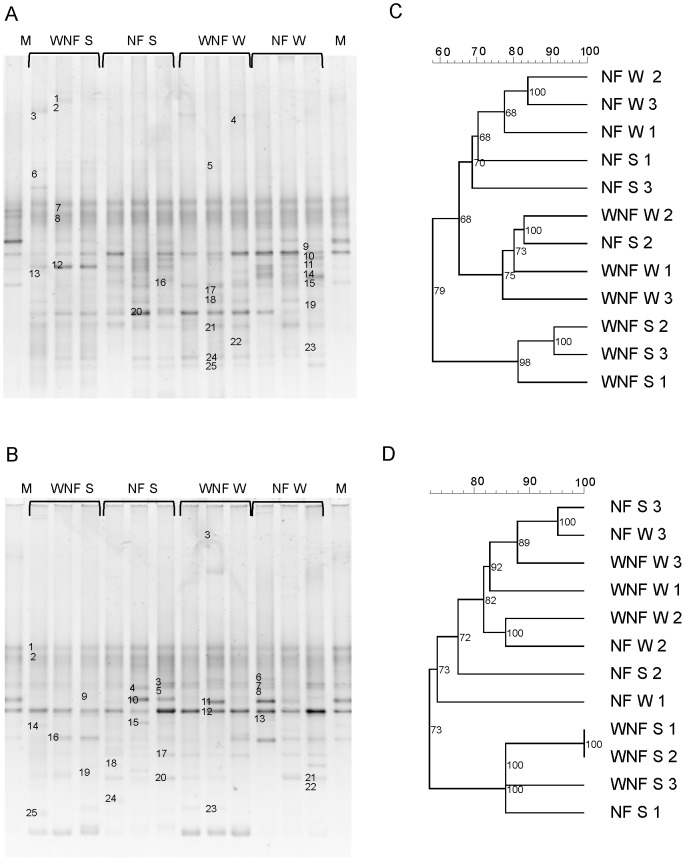
Analysis of the neighbour-joining cluster obtained from the DGGE banding patterns of the microbial communities in soils of *E. globulus* plantations in the Odemira region of southern Portugal. **A**) DGGE band pattern of soil samples using the *nifH* gene. **B**) DGGE band pattern of the root system samples using the *nifH* gene. **C**) Dendrogram of soil samples. **D**) Dendrogram of root system samples. NF: with nitrogen maintenance fertilization; WNF: without nitrogen maintenance fertilization; W: winter; S: summer; M: Marker. Scale  =  similarity (%).

The *nifH* gene was successfully amplified, thus enabling analysis of the amplicon by DGGE. This technique allowed for the culture-independent analysis of DB from the soil and root of the sampled plots (Figures 1, 2 and 3).

The band patterns observed in the DGGE gels were similar between studied plots. Intense bands were predominant in the centres of the gels of all samples; however, it was possible to identify distinct bands, with some appearing more abundant than others (Figures 1–3A and B).

Dendrogram analysis (Figures 1–3C and D) showed that the DB were grouped primarily according to season analysed. However, DB in some soil and root system samples were also grouped by nitrogen fertilization.

In the soils from Penafiel, the DB communities varied more with season than according to N fertilization. Samples taken from the both plots (with and without maintenance N fertilization) in winter displayed 78% similarity (Figure 1C). However, root system samples contained two major groups with 72% similarity according to N fertilization (Figure 1D): the first group which had received maintenance N, with 76% similarity; and the second, which did not receive maintenance N, with 73% similarity (Figure 1D).

Season had the strongest impact on DB communities in both the soil and root systems from Gavião (Figures 2C and 2D). In the soil, three main groups of DB communities were observed, with 74% similarity. Those collected in the summer showed 80% similarity (Figure 2C). Samples of the root systems, showed two main groups of DB communities, with 68% similarity: the first group of the summer samples exhibited 75% similarity; and the second group the winter samples, with 70% similarity (Figure 2D).

Soil samples from Odemira contained three main groups of DB with 58% similarity (Figure 3C); the first showed about 70% similarity and was grouped based on nitrogen fertilization. The season was also a determining factor of the DB communities in soils that had not received N maintenance fertilization: in the summer the DB community profile was very distinct, forming a group with 80% similarity, while in the winter the DB formed a group with 76% similarity (Figure 3C). In the root system the DB community profile formed two major groups with 72% similarity, the first group had 74% similarity and consisted primarily of winter samples, and the second group had 86% similarity and consisted only of summer samples (Figure 3D). In this region, the communities of DB in both soil and root systems of the plot that did not receive maintenance N, were grouped with more than 80% similarity in the summer.

### Effect of environmental factors on diazotrophic bacteria communities

**Figure 4:**
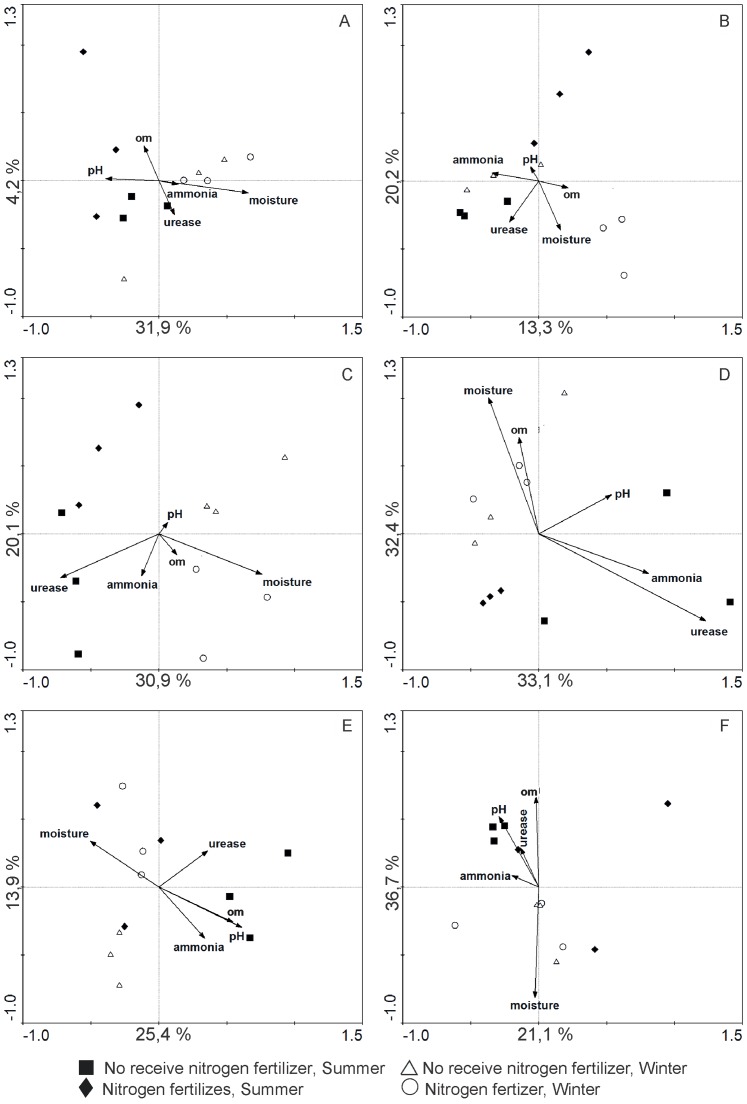
PCA based on PCR-DGGE profiles of the *nifH* gene from soil and root system samples from *E. globulus* plantations in Penafiel (northern), Gavião (central) and Odemira (southern) regions of Portugal, overlaid with environmental data. A and B – soil and root systems (Penafiel); C and D – soil and root systems (Gavião); E and F – soil and root systems (Odemira); om -Organic matter.

Multivariate analysis demonstrated that in the soils from Penafiel, there was an explanation of 36.1% of variation in the DB community (Figure 4A). The first axis explained 31.9% of the variability, and the differentiation in the microbial community was primarily associated with the season, winter and summer (Figure 4A). The DB communities in the summer samples were more closely related to soil pH and organic matter, whereas those in the winter samples showed a higher relation with soil moisture.

In the root system samples, there was an explanation of 33.5% of variation in DB communities, and the groups were primarily separated by nitrogen fertilization (Figure 4B). The samples from plots that did not receive maintenance N presented high relation with ammonia and urease activity (Figure 4B). However, the DB communities in winter samples from plots that received maintenance N fertilization were more affected by soil moisture, while those in summer samples were highly related with soil pH (Figure 4B).

In soils and root systems from the Gavião region, DB communities were grouped according to the seasons (Figures 4C and 4D). Independent of nitrogen maintenance fertilization, winter samples were strongly affected by soil moisture, while those in summer samples from plots without maintenance fertilization were more influenced by urease activity and ammonia. The DB communities in soil and root systems, respectively, 51% and 65.5% of the variations could be explained (Figures 4C and 4D).

In the Odemira region 39.3% of the variability in DB communities in the soils could be explained by the factors considered principally moisture. They were grouped according to nitrogen fertilization (Figure 4E). In samples from plots that did not receive maintenance N fertilization, pH, organic matter and ammonia content were the factors that were more related with the variation in DB communities' structure. The DB community in the root system 57.8% of the variation could be explained and was grouped according to season (Figure 4F). A strong relation between DB communities' structure and moisture was observed in winter samples, whereas a strong relation with pH, urease and organic matter was observed in summer samples (Figure 4F).

### Identification of diazotrophic bacteria communities

**Figure 5:**
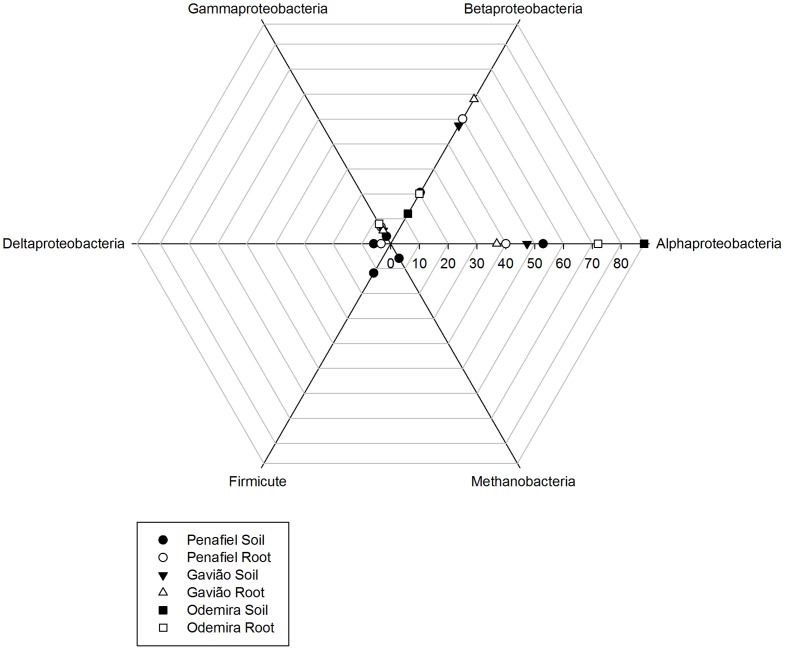
Distribution of predominant bacterial groups in the soils and root systems of *E. globulus* plantations in three regions of Portugal (Penafiel, Gavião and Odemira). Bacteria were identified by band sequencing of the *nifH* gene obtained using the DGGE technique.

The DGGE gel bands of DB from the three regions were sequenced, and the results were compared using the BLASTx algorithm (NCBI). Many bands collected from different positions in the gel were identified as belonging to the same species and most of the bands corresponded to bacteria of the phylum Proteobacteria (Figure 5).

Band sequencing indicated that soil from the Penafiel region contained more species of N-fixing bacteria than samples from the other regions (Figure 5). The phyla Firmicutes (classes Bacilli and Clostridia), Proteobacteria (Deltaproteobacteria class) and also Archaea (Methanobacteria class) were only observed in soil from the Penafiel region (Figure 5). The Deltaproteobacteria class was also observed in root systems from this region (Figure 5).

**Figure 6:**
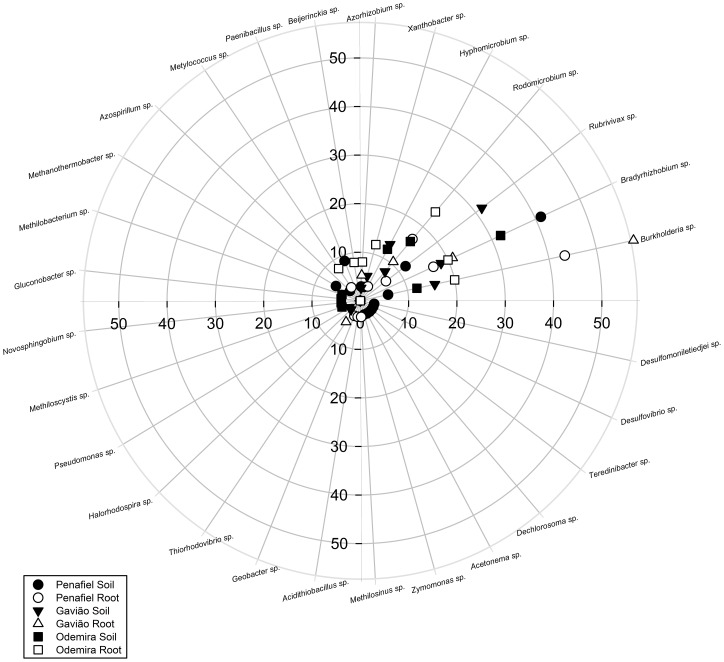
Distribution of bacterial genera found in the soil and root systems of *E. globulus* plantations in three regions of Portugal (Penafiel, Gavião and Odemira) obtained by sequencing the *nifH* gene DGGE bands.

Alphaproteobacteria predominated in the soils from the Odemira and Penafiel whereas there was co-predominance of both Alphaproteobacteria and Betaproteobacteria in soils from the Gavião. Betaproteobacteria predominated in root systems from the Penafiel and Gavião while Alphaproteobacteria predominated in those from the Odemira (Figures 5 and 6). Gammaproteobacteria were less abundant than other classes in samples from all regions (Figure 5 and 6).

The genera *Bradyrhizobium* and *Burkholderia* predominated in the soil and root system samples from Penafiel. In samples from Gavião, the genera *Rubrivivax*, *Bradyrhizobium* and *Burkholderia* were predominant in the soil, while the genus *Burkholderia* predominated in the root system. In samples from the Odemira plantation, the soil contained primarily the genera *Bradyrhizobium* and *Rhodomicrobium*, both of which are Alphaproteobateria, while the root system contained mainly *Rhodomicrobium*, *Bradyrhizobium* and *Burkholderia* (Figure 6).

Of the bacteria belonging to the class Alphaproteobacteria were found in the three regions, 25.1% were of the genus *Bradyrhizobium* and 11.7% of *Rhodomicrobium*. Within the class Betaproteobacteria, the most common genera were *Burkholderia* (23.4%) and *Rubrivivax* (10.5%).

## Discussion

The productivity of a eucalypt forest can be affected by various biotic and abiotic factors, including soil microbial populations and their interactions with the plants. The soil microbial dynamics of these forests and in particular DB communities are relevant due to the considerable importance of eucalypts for industry, economy and the environment. DB communities may serve as a source of reactive N and plant growth promoters [25], including phytohormones, phosphate solubilizers and antagonists of phytopathogens [26].

The differences observed in organic matter, pH, moisture, urease activity and ammonia concentrations in soils of *E. globulus* forests were primarily due to geographic localization and the typical Mediterranean climate, which is characterized by moderately cold, rainy winters and hot, dry summers (Table 1).

The low nitrification potential detected is typical of an acid soil (pH 5, Table 1). Plants are therefore dependent on the microorganisms that act as potential and active reservoirs of N in the soil, and control N availability via immobilization and mineralization processes [25].

As observed in other regions with markedly different seasons [17]; [27], no differences in the total culturable DB were observed between summer and winter (Table 3). However, the structure of the DB community was much influenced firstly by the season and secondly by additional N fertilization (Figures 1, 2 and 3).

PCA indicated that the influence of season on the structure of the DB community was mostly due to soil moisture. PCA was also able to differentiate between the winter and summer samples (Figure 4). This result makes sense because water is essential for cell metabolism in all living organisms. According to Kozdroj and Van Elsas [28] and Baudoin et al. [29], the profile of species in the rhizosphere or plant root system can be affected by various environmental factors, including root exudates and the availability of water and nutrients.

Nitrogen fertilization decreased both the richness and the intensity of the DGGE bands of the DB [24]. It is possible that N fertilization changed the DB community in our study, but the four-year interval between the last fertilization event and sample collection may have diminished the effects of fertilization.

The band pattern observed in the DGGE gels, including the overlapping of various bands across the gel, may reflect the large number of bacterial species common to all the studied regions (Table S1, S2 and S3). However, the band pattern also reflects the amplification of the most abundant species by the nested-PCR, as well as non-detection of species less abundant in the gel [10].

We found the principal bacterial community present in the eucalyptus forests to be Proteobacteria (Figure 5), one of the most diverse, which comprises various classes that contain at least one species of N-fixing bacteria [30]. Additionally, we observed bands that were at different positions in the gel but belong to the same species (Figures 1, 2 and 3, Table S1, S2 and S3). This may be due to the presence of multiple copies of the *nifH* gene in the genome of DB [24], [31], [32]. This gene was selected as marker for DB for its presence in the vast majority of N-fixing bacteria, because it displays highly conserved regions, and since its phylogeny has shown to be consistent with the phylogeny of DB based on the 16S ribosomal RNA gene [31]. The database of the genes that encode for the nitrogenase enzyme is currently one of the largest non-ribosomal gene databases for nonculturable microorganisms [31].

*Bradyrhizobium* and *Burkholderia* (Figure 6), which were also identified in eucalypt plantations in Colombia [17], were the dominant genera of DB in the studied Portuguese eucalypt forests. The genus *Bradyrhizobium* is characterized by moderate adaptation to semi-arid conditions, a propensity to colonize the roots of many non-leguminous plants and the ability to fix nitrogen under both symbiotic and non-symbiotic conditions [33], [34]. The genus *Burkholderia* includes free-living endophytic bacteria that are able to fix nitrogen and bacteria that form symbiotic associations with leguminous plants [35], [36]. Furthermore, the genus *Burkholderia* is able to promote plant growth in different ways, including by solubilizing phosphate, producing siderophores and indole compounds and acting as biocontrol agents [36], [37].

## Conclusions

Culturable methods did not reveal differences in the numbers of DB between the sampling regions or N-fertilization. The profile of the N-fixing bacterial communities was influenced by seasons and nitrogen fertilization regime. The most abundant groups of DB were affiliated to Alpha- and Betaproteobacteria, the main genera being *Burkholderia* and *Bradyrhizobium*. This is the first study of these bacteria in Portuguese eucalypt plantations, and its results contribute to understanding of the specific requirements for the cultivation of these plants.

## Supporting Information

Table S1 **Identification based on the** ***nifH*** **gene of bands eluted from DGGE gels of soil and root system samples from the Penafiel region.**
(DOC)

Table S2 **Identification based on the** ***nifH*** **gene of bands eluted from DGGE gels of soil and root system samples from the Gavião region.**
(DOC)

Table S3 **Identification based on the** ***nifH*** **gene of bands eluted from DGGE gels of soil and root system samples from the Odemira region.**
(DOC)
