# Supplementary material for: Nitrogen-Fixing Bacteria in Eucalyptus globulus Plantations
Source: PLoS One. 2014 Oct 23;9(10):e111313. doi: 10.1371/journal.pone.0111313 (PMC4207822; doi:10.1371/journal.pone.0111313)
Supplement: Table S1 — Identification based on the nifH gene of bands eluted from DGGE gels of soil and root system samples from the Penafiel region. (DOC) [file pone.0111313.s001.doc]

| Banding | Acess gen bank | Identification | Identity (%) | | Similarity (%) | | Class/Order |
| --- | --- | --- | --- | --- | --- | --- | --- |
| **Soil** | | | | | | | |
| 1 ^a^ | [YP_006450330.1](http://www.ncbi.nlm.nih.gov/protein/392413723?report=genbank&log$=prottop&blast_rank=1&RID=2N542JYP01N) | Mo-nitrogenase iron protein subunit NifH: *Desulfomonile tiedje*i | | 82 | 89 | Deltaproteobacteria; Syntrophobacterales | |
| 7 ^a^, 2 ^b^ | [ZP_09772528.1](http://www.ncbi.nlm.nih.gov/protein/375307239?report=genbank&log$=prottop&blast_rank=1&RID=2N5G0HX301N) | Nitrogenase iron protein subunit NifH*:* *Paenibacillus* sp. | | 61 - 86 | 74 - 90 | Bacilli; Bacillales; | |
| 3 ^a^ | [YP_003226535.1](http://www.ncbi.nlm.nih.gov/protein/260753642?report=genbank&log$=prottop&blast_rank=1&RID=2N5PBZS801S) | Nitrogenasereductase: *Zymomonas mobilis* sp. | | 59 | 69 | Alphaproteobacteria; Sphingomonadales | |
| 4, 27 | [ZP_09750647.1](http://www.ncbi.nlm.nih.gov/protein/375104386?report=genbank&log$=prottop&blast_rank=1&RID=2N5TS9SH01N) | Nitrogenase iron protein: Burkholderiales bacterium | | 93 - 94 | 96 - 98 | Betaproteobacteria; Burkholderiales | |
| 5, 8, 9, 10 | [YP_005439123.1](http://www.ncbi.nlm.nih.gov/protein/383760137?report=genbank&log$=prottop&blast_rank=1&RID=2N5WRUGU01S) | Nitrogenase iron protein NifH: *Rubrivivax gelatinosus* | | 87 - 92 | 93 - 98 | Betaproteobacteria; Burkholderiales | |
| 6 | [YP_005075592.1](http://www.ncbi.nlm.nih.gov/protein/374322463?report=genbank&log$=prottop&blast_rank=1&RID=2N60VJNU013) | nitrogenase iron protein subunit NifH: *Paenibacillus terrae* | | 82 | 88 | Bacilli; Bacillales; | |
| 11 | [YP_001526359.1](http://www.ncbi.nlm.nih.gov/protein/158425067?report=genbank&log$=prottop&blast_rank=1&RID=3729H5YG014) | nifH gene product: *Azorhizobium caulinodans* | | 91 | 93 | Alphaproteobacteria; Rhizobiales | |
| 12 ^a^ | [YP_005451908.1](http://www.ncbi.nlm.nih.gov/protein/383772842?report=genbank&log$=prottop&blast_rank=1&RID=2NDN8F4C013) | Nitrogenase iron protein: *Bradyrhizobium* sp. | | 96 | 100 | Alphaproteobacteria; Rhizobiales | |
| 13 ^a^ | [ZP_08626728.1](http://www.ncbi.nlm.nih.gov/protein/338814742?report=genbank&log$=prottop&blast_rank=1&RID=2NDXUP3X016) | Nitrogenase: *Acetonema longum* | | 56 | 68 | Clostridia; Selenomonadales | |
| 15, 14 ^b^ | [YP_003849930.1](http://www.ncbi.nlm.nih.gov/protein/304314783?report=genbank&log$=prottop&blast_rank=1&RID=2P3JH96Y01S) | Nitrogenase iron protein: *Methanothermobacter*  *marburgensis* | | 72 - 88 | 82 - 83 | Methanobacteria; Methanobacteriales | |
| 16 ^a^, 17 ^a^,18 ^b^, 19, 20, 21^b^, 23 ^b^, 24 ^a^, 25 ^a^, 30 ^a^, 31 ^a^, 34 ^a^ | [ZP_09436926.1](http://www.ncbi.nlm.nih.gov/protein/365899001?report=genbank&log$=prottop&blast_rank=1&RID=2P463PTF01S) | fragment of nitrogenase iron protein: *Bradyrhizobium* sp. | | 63 - 97 | 75 - 100 | Alphaproteobacteria; Rhizobiales | |
| 22 | [NP_768409.1](http://www.ncbi.nlm.nih.gov/protein/27376880?report=genbank&log$=prottop&blast_rank=1&RID=2PJEY349016) | *nifH* gene product: *Bradyrhizobium japonicum* | | 88 | 94 | Alphaproteobacteria; Rhizobiales | |
| 26 | [YP_005027201.1](http://www.ncbi.nlm.nih.gov/protein/372487636?report=genbank&log$=prottop&blast_rank=1&RID=2PK983RY013) | Nitrogenase iron protein: *Dechlorosoma suillum* | | 92 | 93 | Betaproteobacteria; Rhodocyclales | |
| 28 | [ZP_08870627.1](http://www.ncbi.nlm.nih.gov/protein/347739340?report=genbank&log$=prottop&blast_rank=1&RID=2PKG49MM013) | Nitrogenase reductase: *Azospirillum amazonense* | | 79 | 73 | Alphaproteobacteria; Rhodospirillales | |
| 29 | [YP_003073074.1](http://www.ncbi.nlm.nih.gov/protein/254785645?report=genbank&log$=prottop&blast_rank=1&RID=2PKNAE8B013) | nitrogenase iron protein: *Teredinibacter turnerae* | | 61 | 75 | Gammaproteobacteria; Alteromonadales; | |
| 32 ^b^ | [YP_001415059.1](http://www.ncbi.nlm.nih.gov/protein/154244101?report=genbank&log$=prottop&blast_rank=1&RID=2PM4832W01S) | Nitrogenase reductase: *Xanthobacter autotrophicus* | | 71 | 80 | Alphaproteobacteria; Rhizobiales | |
| 33 ^a^ | [ZP_10077601.1](http://www.ncbi.nlm.nih.gov/protein/386392820?report=genbank&log$=prottop&blast_rank=1&RID=2PM73VBN013) | Nitrogenase iron protein: *Desulfovibrio* sp*.* | | 74% | 81 | Deltaproteobacteria; Desulfovibrionales | |
| **Root system** | | | | | | | |
| 1, 6, 8,10,12 | [YP_004012288.1](http://www.ncbi.nlm.nih.gov/protein/312114692?report=genbank&log$=prottop&blast_rank=1&RID=8FRFKUHE01R) | Nitrogenase iron protein: *Rhodomicrobium vannielii* | | 88-94 | 95 - 99 | Alphaproteobacteria; Rhizobiales | |
| 2 | [ZP_10031712.1](http://www.ncbi.nlm.nih.gov/protein/385204842?report=genbank&log$=prottop&blast_rank=1&RID=8FRJZKHD01R) | Nitrogenase iron protein: *Burkholderia* sp. | | 97 | 97 | Betaproteobacteria; Burkholderiales | |
| 3, 9, 15,16^b^,18,19,  20, 21, 22, 25 | [YP_553849.1](http://www.ncbi.nlm.nih.gov/protein/91778641?report=genbank&log$=prottop&blast_rank=3&RID=8FRP19YC01R) | Nitrogenase reductase: *Burkholderia xenovorans* | | 82-95 | 83 - 97 | Betaproteobacteria; Burkholderiales | |
| 4^b^ | [ZP_06886595.1](http://www.ncbi.nlm.nih.gov/protein/296444631?report=genbank&log$=prottop&blast_rank=1&RID=8FUBVB30016) | Nitrogenase iron protein: *Methylosinus trichosporium* | | 82 | 92 | Alphaproteobacteria; Rhizobiales | |
| 5 ^b^, 7,13,17,28 | [YP_005451908.1](http://www.ncbi.nlm.nih.gov/protein/383772842?report=genbank&log$=prottop&blast_rank=1&RID=8FUGDRV4014) | Nitrogenase iron protein: *Bradyrhizobium* sp. | | 84-95 | 92 - 100 | Alphaproteobacteria; Rhizobiales | |
| 11 | [ZP_08402608.1](http://www.ncbi.nlm.nih.gov/protein/332526496?report=genbank&log$=prottop&blast_rank=2&RID=8FUVV2HJ01R) | Nitrogenase reductase: *Rubrivivax benzoatilyticus* | | 83 | 92 | Betaproteobacteria; Burkholderiales | |
| 14 | [YP_005439123.1](http://www.ncbi.nlm.nih.gov/protein/383760137?report=genbank&log$=prottop&blast_rank=1&RID=8FV3DJ4V014) | Nitrogenase iron protein NifH: *Rubrivivax gelatinosus* | | 90 | 97 | Betaproteobacteria; Burkholderiales | |
| 23 | [YP_004677463.1](http://www.ncbi.nlm.nih.gov/protein/338740501?report=genbank&log$=prottop&blast_rank=1&RID=8G7UPCRK014) | Nitrogenase iron protein: *Hyphomicrobium* sp. | | 94 | 96 | Alphaproteobacteria; Rhizobiales | |
| 24 | [YP_004784109.1](http://www.ncbi.nlm.nih.gov/protein/344199783?report=genbank&log$=prottop&blast_rank=1&RID=8G7Y7UGS014) | Nitrogenase iron protein: *Acidithiobacillus ferrivorans* | | 86 | 76 | Gammaproteobacteria; Acidithiobacillales; | |
| 26 | [ZP_09750647.1](http://www.ncbi.nlm.nih.gov/protein/375104386?report=genbank&log$=prottop&blast_rank=1&RID=8G8BGKXP014) | Nitrogenase iron protein: Burkholderiales bacterium | | 96 | 97 | Betaproteobacteria; Burkholderiales | |
| 27 ^b^ | [YP_112764.1](http://www.ncbi.nlm.nih.gov/protein/53802581?report=genbank&log$=prottop&blast_rank=1&RID=8G8T93TA01R) | Nitrogenase reductase: *Methylococcus capsulatus* | | 90 | 92 | Gammaproteobacteria; Methylococcales | |
| 29^b^ | [YP_003610238.1](http://www.ncbi.nlm.nih.gov/protein/295701237?report=genbank&log$=prottop&blast_rank=2&RID=8GBP4F7A01R) | Nitrogenase iron protein: *Burkholderia* sp. | | 93 | 97 | Betaproteobacteria; Burkholderiales | |
| 30^b^ | [YP_001229952.1](http://www.ncbi.nlm.nih.gov/protein/148263246?report=genbank&log$=prottop&blast_rank=1&RID=8GBWZXFM01R) | Nitrogenase iron protein: *Geobacter uraniireducens* | | 87 | 100 | Deltaproteobacteria; Desulfuromonadales | |
| ^a^  Only primer 278R, ^b^ Only primer 19F | | | |  |  |  | |

**Table S1 –** Identification based on the *nifH* gene of bands eluted from DGGE gels of soil and root system samples from the Penafiel region.
