# Supplementary material for: Nitrogen-Fixing Bacteria in Eucalyptus globulus Plantations
Source: PLoS One. 2014 Oct 23;9(10):e111313. doi: 10.1371/journal.pone.0111313 (PMC4207822; doi:10.1371/journal.pone.0111313)
Supplement: Table S2 — Identification based on the nifH gene of bands eluted from DGGE gels of soil and root system samples from the Gavião region. (DOC) [file pone.0111313.s002.doc]

**Table S2 –** Identification based on the *nifH* gene of bands eluted from DGGE gels of soil and root system samples from the Gavião region.

| Banding | Acess gen bank | | Identification | | Identity (%) | | Similarity (%) | | | Class/Order |
| --- | --- | --- | --- | --- | --- | --- | --- | --- | --- | --- |
| **Soil** | | | | | | | | | | |
| 1, 2, 21, 23, 24 | | [YP_004677463.1](http://www.ncbi.nlm.nih.gov/protein/338740501?report=genbank&log$=prottop&blast_rank=1&RID=1ZMFSYAP016) | | *nifH* gene product: *Hyphomicrobium* sp. | | 90 - 93 | | 96 - 98 | Alphaproteobacteria; Rhizobiales | |
| 3, 4, 5, 6, 9, 11, 12,  13, 14, 16, 17, 18 | | [YP_005439123.1](http://www.ncbi.nlm.nih.gov/protein/383760137?report=genbank&log$=prottop&blast_rank=1&RID=224CTWH9016) | | Nitrogenase iron protein NifH: *Rubrivivax gelatinosus* | | 90 - 92 | | 97 - 98 | Betaproteobacteria; Burkholderiales | |
| 7, 19, 22, 26 | | [ZP_09750647.1](http://www.ncbi.nlm.nih.gov/protein/375104386?report=genbank&log$=prottop&blast_rank=1&RID=227VNR2401N) | | Nitrogenase iron protein: Burkholderiales bacterium | | 91 - 96 | | 96 - 100 | Betaproteobacteria; Burkholderiales | |
| 8 | | [ZP_09436926.1](http://www.ncbi.nlm.nih.gov/protein/365899001?report=genbank&log$=prottop&blast_rank=1&RID=22FS3G3S014) | | fragment of nitrogenase iron protein, NifH: *Bradyrhizobium* sp. | | 92 | | 98 | Alphaproteobacteria; Rhizobiales | |
| 10, 27^b^ | | [YP_005451908.1](http://www.ncbi.nlm.nih.gov/protein/383772842?report=genbank&log$=prottop&blast_rank=1&RID=22GDUEAG014) | | Nitrogenase iron protein: *Bradyrhizobium* sp. | | 91 - 92 | | 96 - 99 | Alphaproteobacteria; Rhizobiales | |
| 15 | | [ZP_10031712.1](http://www.ncbi.nlm.nih.gov/protein/385204842?report=genbank&log$=prottop&blast_rank=1&RID=28DTUW2K012) | | Nitrogenase iron protein: *Burkholderia* sp. | | 97 | | 97 | Betaproteobacteria; Burkholderiales | |
| 20 | | [YP_001523957.1](http://www.ncbi.nlm.nih.gov/protein/158422665?report=genbank&log$=prottop&blast_rank=3&RID=2S27XD8R01S) | | *nifH* gene product: *Azorhizobium caulinodans* | | 92 | | 97 | Alphaproteobacteria; Rhizobiales | |
| 25 ^b^ | | [YP_553849.1](http://www.ncbi.nlm.nih.gov/protein/91778641?report=genbank&log$=prottop&blast_rank=1&RID=2G5ZNEMT013) | | *nifH* gene product: *Burkholderia xenovorans* | | 92 | | 96 | Betaproteobacteria; Burkholderiales | |
| 31^a^, 36^a^, 38^a^ | | [YP_004012288.1](http://www.ncbi.nlm.nih.gov/protein/312114692?report=genbank&log$=prottop&blast_rank=1&RID=2G66A8FP01S) | | Nitrogenase iron protein: *Rhodomicrobium vannielii* | | 97 | | 98 - 99 | Alphaproteobacteria; Rhizobiales | |
| 28, 32^a^ | | [ZP_09436926.1](http://www.ncbi.nlm.nih.gov/protein/365899001?report=genbank&log$=prottop&blast_rank=1&RID=2G76DCR0013) | | fragment of nitrogenase iron protein, NifH: *Bradyrhizobium* sp. | | 94 - 98 | | 97 - 98 | Alphaproteobacteria; Rhizobiales | |
| 29,30^a^ | | [YP_001415059.1](http://www.ncbi.nlm.nih.gov/protein/154244101?report=genbank&log$=prottop&blast_rank=1&RID=2G7CWMJ801S) | | Nitrogenase reductase: *Xanthobacter autotrophicus* | | 87 - 89 | | 92 - 93 | Alphaproteobacteria; Rhizobiales | |
| 33^a^ | | [YP_001171863.1](http://www.ncbi.nlm.nih.gov/protein/146281710?report=genbank&log$=prottop&blast_rank=1&RID=2G8TF8E901N) | | Fe protein, nitrogenase reductase NifH*: Pseudomonas stutzeri* | | 83 | | 89 | Gammaproteobacteria; Pseudomonadales | |
| 34, 35^a^ | | [YP_005451908.1](http://www.ncbi.nlm.nih.gov/protein/383772842?report=genbank&log$=prottop&blast_rank=1&RID=2G8XDKTT01S) | | Nitrogenase iron protein: *Bradyrhizobium* sp. | | 89 - 99 | | 94 -100 | Alphaproteobacteria; Rhizobiales | |
| 37 | | [YP_001001870.1](http://www.ncbi.nlm.nih.gov/protein/121997083?report=genbank&log$=prottop&blast_rank=2&RID=3D7YXBH8016) | | Nitrogenase iron protein: *Halorhodospira halophila* | | 88 | | 89 | Gammaproteobacteria; Chromatiales | |
| **Root system** | | | | | | | | | | |
| 1,2,5 | | [YP_553849.1](http://www.ncbi.nlm.nih.gov/protein/91778641?report=genbank&log$=prottop&blast_rank=1&RID=8J3XA5SN014) | | Nitrogenase reductase: *Burkholderia xenovorans* | | 90 - 98 | | 93 - 98 | Betaproteobactérias; Burkholderiales | |
| 3,7 | | [YP_004012288.1](http://www.ncbi.nlm.nih.gov/protein/312114692?report=genbank&log$=prottop&blast_rank=3&RID=8J40BTKR014) | | Nitrogenase iron protein: *Rhodomicrobium vannielii* | | 92 - 93 | | 97 - 98 | Alphaproteobacteria; Rhizobiales | |
| 4 | | [ZP_10031712.1](http://www.ncbi.nlm.nih.gov/protein/385204842?report=genbank&log$=prottop&blast_rank=3&RID=8J423NNH016) | | Nitrogenase iron protein: *Burkholderia* sp. | | 93 | | 95 | Betaproteobactérias; Burkholderiales | |
| 6, 8,9, 11,14,16,19 | | [ZP_09750647.1](http://www.ncbi.nlm.nih.gov/protein/375104386?report=genbank&log$=prottop&blast_rank=1&RID=8J45PZS5014) | | Nitrogenase iron protein: *Burkholderiale*s | | 90 - 98 | | 94 - 98 | Betaproteobactérias; Burkholderiales | |
| 10 | | [ZP_09812602.1](http://www.ncbi.nlm.nih.gov/protein/377661373?report=genbank&log$=prottop&blast_rank=2&RID=8J4H5FW401R) | | Nitrogenase iron protein: *Thiorhodovibrio* sp. | | 80 | | 95 | Gammaproteobacteria; Chromatiales | |
| 12^b^,17, 18 | | [YP_005451908.1](http://www.ncbi.nlm.nih.gov/protein/383772842?report=genbank&log$=prottop&blast_rank=3&RID=8J4YM34A014) | | Nitrogenase iron protein: *Bradyrhizobium* sp. | | 92-99 | | 96-100 | Alphaproteobacteria; Rhizobiales | |
| 13 | | [YP_001523957.1](http://www.ncbi.nlm.nih.gov/protein/158422665?report=genbank&log$=prottop&blast_rank=2&RID=8J50E2PM016) | | Nitrogenase reductase: *Azorhizobium caulinodans* | | 94 | | 96 | Alphaproteobacteria; Rhizobiales | |
| 15 | | [ZP_09436926.1](http://www.ncbi.nlm.nih.gov/protein/365899001?report=genbank&log$=prottop&blast_rank=1&RID=8J54Y2H0014) | | fragment of nitrogenase iron protein, NifH: *Bradyrhizobium* sp. | | 95 | | 96 | Alphaproteobacteria; Rhizobiales | |

^a^ Only primer 278R, ^b^ Only primer 19F
