# Supplementary material for: Nitrogen-Fixing Bacteria in Eucalyptus globulus Plantations
Source: PLoS One. 2014 Oct 23;9(10):e111313. doi: 10.1371/journal.pone.0111313 (PMC4207822; doi:10.1371/journal.pone.0111313)
Supplement: Table S3 — Identification based on the nifH gene of bands eluted from DGGE gels of soil and root system samples from the Odemira region. (DOC) [file pone.0111313.s003.doc]

**Table S3 –** Identification based on the *nifH* gene of bands eluted from DGGE gels of soil and root system samples from the Odemira region.

| Banding | Acess gen bank | | Identification | | Identity (%) | | Similarity (%) | | | Class/Order |
| --- | --- | --- | --- | --- | --- | --- | --- | --- | --- | --- |
| **Soil** | | | | | | | | | | |
| 1^a^, 2^a^, 13^a^ | | [ZP_09436926.1](http://www.ncbi.nlm.nih.gov/protein/365899001?report=genbank&log$=prottop&blast_rank=1&RID=2RN8JUYA013) | | Nitrogenase iron protein, NifH*:* *Bradyrhizobium* sp | | 77 - 95 | | 86 - 96 | Alphaproteobacteria; Rhizobiales | |
| 3^b^ | | [YP_001770351.1](http://www.ncbi.nlm.nih.gov/protein/170741696?report=genbank&log$=prottop&blast_rank=1&RID=2RHK7FH201N) | | *nifH* gene product Methylobacterium sp. | | 77 % | | 85 % | Alphaproteobacteria; Rhizobiales | |
| 4, 5, 8^a^,14^a^ | | [YP_004012288.1](http://www.ncbi.nlm.nih.gov/protein/312114692?report=genbank&log$=prottop&blast_rank=1&RID=8JCM22W1014) | | Nitrogenase iron protein: *Rhodomicrobium vannielii* | | 78 - 97 | | 87 -100 | Alphaproteobacteria; Rhizobiales | |
| 6 | | [YP_001600720.1](http://www.ncbi.nlm.nih.gov/protein/162146261?report=genbank&log$=prottop&blast_rank=1&RID=2RM98ZJX01S) | | Nitrogenasereductase: *Gluconacetobacter diazotrophicus* | | 88 | | 96 | Alphaproteobacteria; Rhodospirillales | |
| 7,9 ^b^ | | [YP_005451950.1](http://www.ncbi.nlm.nih.gov/protein/383772884?report=genbank&log$=prottop&blast_rank=3&RID=8GF68UDG01R) | | Nitrogenase iron protein: *Bradyrhizobium* sp*.* | | 85 - 93 | | 95 | Alphaproteobacteria; Rhizobiales | |
| 10, 22 ^b^ | | [P_001415059.1](http://www.ncbi.nlm.nih.gov/protein/154244101?report=genbank&log$=prottop&blast_rank=1&RID=8GJMJU1G016) | | Nitrogenase reductase: *Xanthobacter autotrophicus* | | 88 -100 | | 98 -100 | Alphaproteobacteria; Rhizobiales | |
| 11 | | [ZP_10031712.1](http://www.ncbi.nlm.nih.gov/protein/385204842?report=genbank&log$=prottop&blast_rank=5&RID=8GM15U6U01R) | | Nitrogenase iron protein: *Burkholderia* sp. | | 94 | | 96 | Betaproteobactérias; Burkholderiales | |
| 12^a^, 25 | | [YP_005451908.1](http://www.ncbi.nlm.nih.gov/protein/383772842?report=genbank&log$=prottop&blast_rank=3&RID=8GFH3406016) | | Nitrogenase iron protein: *Bradyrhizobium* sp. | | 97 - 98 | | 98 | Alphaproteobacteria; Rhizobiales | |
| 15 | | [YP_001241772.1](http://www.ncbi.nlm.nih.gov/protein/148257187?report=genbank&log$=prottop&blast_rank=1&RID=8JAKZ66T01R) | | Nitrogenase reductase: *Bradyrhizobium* sp. | | 95 | | 100 | Alphaproteobacteria; Rhizobiales | |
| 16^a^ | | [ZP_08207527.1](http://www.ncbi.nlm.nih.gov/protein/326385902?report=genbank&log$=prottop&blast_rank=1&RID=8GGBUCPB01R) | | Nitrogenase reductase: *Novosphingobium nitrogenifigens* | | 63 | | 69 | Alphaproteobacteria; Sphingomonadales | |
| 17,18, 21 | | [YP_004677463.1](http://www.ncbi.nlm.nih.gov/protein/338740501?report=genbank&log$=prottop&blast_rank=1&RID=8GGJYWG901R) | | Nitrogenase iron protein 2: *Hyphomicrobium* sp. | | 95 -100 | | 97 -100 | Alphaproteobacteria; Rhizobiales | |
| 19, 20 | | [ZP_09750647.1](http://www.ncbi.nlm.nih.gov/protein/375104386?report=genbank&log$=prottop&blast_rank=1&RID=8GG714YF01R) | | Nitrogenase iron protein: Burkholderiales bacterium | | 77 - 96 | | 82 - 96 | Betaproteobactérias; Burkholderiales | |
| 23 | | [YP_006592725.1](http://www.ncbi.nlm.nih.gov/protein/402773188?report=genbank&log$=prottop&blast_rank=1&RID=8JB3327U016) | | Nitrogenase iron protein 2,NifH: *Methylocystis* sp. | | 94 | | 96 | Alphaproteobacteria; Rhizobiales; | |
| 24^b^ | | [YP_001415004.1](http://www.ncbi.nlm.nih.gov/protein/154244046?report=genbank&log$=prottop&blast_rank=1&RID=8GH36USW01R) | | Nitrogenase reductase: *Xanthobacter autotrophicus* | | 94 | | 98 | Alphaproteobacteria; Rhizobiales | |
| **Root system** | | | | | | | | | | |
| 1,17 | | [YP_005451950.1](http://www.ncbi.nlm.nih.gov/protein/383772884?report=genbank&log$=prottop&blast_rank=1&RID=8JB4TR1101R) | | Nitrogenase iron protein: *Bradyrhizobium* sp. | | 90 - 96 | | 97-99 | Alphaproteobacteria; Rhizobiales | |
| 2 | | [YP_005451908.1](http://www.ncbi.nlm.nih.gov/protein/383772842?report=genbank&log$=prottop&blast_rank=1&RID=8JB6XV4N014) | | Nitrogenase iron protein: *Bradyrhizobium* sp. | | 93 | | 95 | Alphaproteobacteria; Rhizobiales | |
| 3,12,14, 15, 24, 21^a^ | | [YP_004012288.1](http://www.ncbi.nlm.nih.gov/protein/312114692?report=genbank&log$=prottop&blast_rank=1&RID=8JB8AATE01R) | | Nitrogenase iron protein: *Rhodomicrobium vannielii* | | 54 - 97 | | 64 -100 | Alphaproteobacteria; Rhizobiales | |
| 4,10 | | [NP_768409.1](http://www.ncbi.nlm.nih.gov/protein/27376880?report=genbank&log$=prottop&blast_rank=1&RID=8JBZCZ4K01R) | | Nitrogenase reductase: *Bradyrhizobium japonicum* | | 86 - 98 | | 88 -100 | Alphaproteobacteria; Rhizobiales | |
| 5, 25 | | [YP_553849.1](http://www.ncbi.nlm.nih.gov/protein/91778641?report=genbank&log$=prottop&blast_rank=3&RID=8JBCV5VM014) | | Nitrogenase reductase: *Burkholderia xenovorans* | | 93 - 94 | | 95 - 96 | Betaproteobactérias; Burkholderiales | |
| 6,8 | | [YP_001831615.1](http://www.ncbi.nlm.nih.gov/protein/182677469?report=genbank&log$=prottop&blast_rank=1&RID=8JDJ21Z5014) | | Nitrogenase reductase: *Beijerinckia indica* | | 90 - 93 | | 100 | Alphaproteobacteria; Rhizobiales | |
| 7, 11 | | [YP_001523957.1](http://www.ncbi.nlm.nih.gov/protein/158422665?report=genbank&log$=prottop&blast_rank=2&RID=8JD6SYFX014) | | Nitrogenase reductase: *Azorhizobium caulinodans* | | 91 | | 96 - 98 | Alphaproteobacteria; Rhizobiales | |
| 9,18,20 | | [ZP_09750647.1](http://www.ncbi.nlm.nih.gov/protein/375104386?report=genbank&log$=prottop&blast_rank=1&RID=8JBRFBXU01R) | | Nitrogenase iron protein: Burkholderiales bacterium | | 94 - 96 | | 97 | Betaproteobactérias; Burkholderiales | |
| 13 | | [YP_001415059.1](http://www.ncbi.nlm.nih.gov/protein/154244101?report=genbank&log$=prottop&blast_rank=1&RID=8JDWKYB6016) | | Nitrogenase reductase: *Xanthobacter autotrophicus* | | 92 | | 97 | Alphaproteobacteria; Rhizobiales; | |
| 16^b^, | | [YP_112764.1](http://www.ncbi.nlm.nih.gov/protein/53802581?report=genbank&log$=prottop&blast_rank=1&RID=8JBGJ3A6014) | | Nitrogenase reductase: *Methylococcus capsulatus* | | 88 | | 88 | Gammaproteobacteria; Methylococcales | |
| 19,23 | | [YP_001415059.1](http://www.ncbi.nlm.nih.gov/protein/154244101?report=genbank&log$=prottop&blast_rank=1&RID=8JDWKYB6016) | | Nitrogenase reductase: *Xanthobacter autotrophicus* | | 92 | | 97 | Alphaproteobacteria; Rhizobiales; | |
| 22^b^ | | [YP_112764.1](http://www.ncbi.nlm.nih.gov/protein/53802581?report=genbank&log$=prottop&blast_rank=1&RID=8JBGJ3A6014) | | Nitrogenase reductase: *Methylococcus capsulatus* | | 88 | | 88 | Gammaproteobacteria; Methylococcales | |
| ^a^ Only primer 278R, ^b^ Only primer 19F | | | | | |  | |  |  | |
